# A Comprehensive Review of Retrofitted Reinforced Concrete Members Utilizing Ultra-High-Performance Fiber-Reinforced Concrete

**DOI:** 10.3390/ma18050945

**Published:** 2025-02-21

**Authors:** Firas Hassan Saeed, Farzad Hejazi

**Affiliations:** Faculty of Environment and Technology, The University of the West England, Bristol BS16 1QY, UK; firashasan43@gmail.com

**Keywords:** ultra-high-performance fiber-reinforced concrete (UHPFRC), strengthening, finite element model, analytical design models, RC structures

## Abstract

Strengthening reinforced concrete (RC) buildings is a critical challenge in the construction industry, pushed by the necessity to address aging infrastructure, environmental degradation, and growing use requirements. Ultra-high-performance fiber-reinforced concrete (UHPFRC) is one of the advanced materials that present a viable solution owing to its exceptional durability and mechanical characteristics, which encompass higher compressive and tensile strengths, low permeability, and resilience against intense environmental as chloride ingress, cycles of freeze–thaw, and chemical assaults. This literature review comprehensively examines UHPFRC as a rehabilitation or strengthening mix material for the RC slabs and beams. Experimental key subjects include the influence of bonding techniques, strengthening configurations, steel fiber ratios, UHPFRC thickness, and reinforcing steel within the UHPFRC layer. In addition, the existing numerical and analytical approaches for forecasting the flexural or shear capability of reinforcing concrete structures retrofitted with UHPFRC were examined and critically assessed. Despite the improvements in the RC structures achieved through experiments utilizing UHPFRC as a reinforcement layer, this study highlights some deficiencies in the existing knowledge, such as the absence of effective ways to address debonding, insufficient research on cyclic loading, and the necessity for economical and sustainable strengthening techniques. This review establishes a basis for future research, intending to create an innovative UHPFRC-based strengthening system that mitigates current limits and improves the overall efficacy, performance, and durability of RC structures.

## 1. Introduction

In recent decades, the principal challenges related to aging and degradation have emerged as significant concerns in the building industry. Reinforced concrete members are designed to withstand various environmental situations during their expected functional lifespan. Nonetheless, human mistakes including shifts in usage, weaknesses in design, insufficient maintenance of members, and changeovers in environmental circumstances could result in considerable deterioration over time [1,2,3]. Due to these challenges, it is necessary to conduct a comprehensive evaluation and find inventive remedies for ensuring the resilience and life span of the structure, and it is imperative to rehabilitate or strengthen existing members [4,5]. Various methods and new materials have been generated during the last three decades to strengthen RC structures, such as concrete/steel jacketing, fiber-reinforced polymers (FRPs), engineering cementitious composites (ECCs), and epoxy injection and grouting [6,7,8,9,10,11,12,13,14]. While these approaches may effectively achieve strengthening objectives and durability, they also present certain drawbacks, including increased structural weight, issues of corrosion, and fire resistance, in addition to the degradation of adhesive materials employed at the interaction face in the FRP [15,16,17,18]. Recently, a new cement-based substance, ultra-high-performance fiber-reinforced concrete (UHPFRC) material, was devised [19,20]. UHPFRC, an innovative building material, has high tensile and compressive strength and outstanding durability, earning it an ideal material for strengthening structures. The basic components for UHPFRC include cement, fume of silica, quartz sand, quartz powder, steel fibers, and superplasticizers [19]. Compared to conventional concrete, UHPFRC exhibits significantly higher compression and tensile strengths (minimum of 140 MPa and 7 MPa, respectively), and enhanced toughness. UHPFRC has a compact micro-structure and low permeability, resulting in the betterment of durability and outstanding resistance to environmental degradation. As a result, UHPFRC shows significant resistance to the overall degradation operations, including the infiltration of carbon dioxide, sulphate, chloride, and corrosive liquids, in addition to the damage from cycles of freeze–thaw and thermal stresses, ensuring long-term durability in extreme environments [21,22,23,24]. Furthermore, the superior rheological characteristics of UHPFRC provide effortless casting in its fresh form [25]. In addition to its mechanical and durability benefits, UHPFRC markedly improves structural safety and resilience, especially in structures subjected to severe situations like earthquakes, impacts, and explosions. Its superior energy absorption and crack-bridging properties augment a structure’s capacity to endure dynamic loads and mitigate the likelihood of serious damage. Integrating UHPFRC into strengthening and retrofitting procedures significantly enhances the service life of existing structures while increasing their overall performance and resilience to extreme environmental and mechanical loads. The use of the UHPFRC material for the repair/rehabilitation of bridges or parts of bridges can be seen in different countries like Switzerland, Canada, The Netherlands, China, France, Malaysia, Japan, and Germany [26,27,28,29]. Moreover, the research and development of UHPFRC advance concrete material science and building technology, promoting innovations in new structural systems. Its exceptional properties authorize the optimization of material efficiency and structural performance, contributing to endurable infrastructure development. By extending the lifespan of existing structures, decreasing maintenance costs, and minimizing the need for full reconstruction, UHPFRC demonstrates a possible and forward-thinking solution for trendy civil engineering challenges. This investigation offers a comprehensive review of the effectiveness of UHPFRC in strengthening RC structures, focusing on slabs and beams. The investigation assesses critical parameters such as bonding technique, strengthening configuration, steel fiber ratio, UHPFRC thickness, reinforced UHPFRC layer, ultimate load, and failure mode. Additionally, numerical and analytical models are reviewed to replicate the structural behavior and load-carrying capacity of RC members strengthened with UHPFRC. By addressing these aspects, this analysis bridges the gap between theoretical studies and experimental applications, illustrating the possibility of UHPFRC as a creative strengthening solution for deteriorated RC structures.

## 2. Definition and Historical Development of UHPFRC

UHPFRC is a developed sort of cementitious composite distinguished by its ultra-high compression strength (often surpassing 140 MPa), higher tensile strength, exceptional durability, and improved ductility resulting from the presence of steel fibers. UHPFRC is recognized for its superior mechanical properties, particularly its tensile strength, which significantly exceeds that of traditional concrete. The tensile behavior of UHPFRC is characterized by its ability to undergo strain hardening, leading to improved ductility and energy absorption capacity [19,20]. UHPFRC generally indicates a direct tensile strength varying from roughly 7 to 10 MPa, depending on the specific mixture design and fiber content [19]. UHPFRC possesses a dense microstructure and low porosity, providing resilience against chemical assaults, freeze–thaw cycles, and environmental deterioration. It is extensively utilized for strengthening, rehabilitation, and building robust infrastructure. UHPFRC can be regarded as an amalgamation of strategies; self-compacted (SCC), fiber-reinforcement (FRC), and the last is high-performing (HPC) [28]. UHPFRC consists of premium ordinary cement, fumes of silica, sand, quartz powder, superplasticizers, very low water, and occasionally supplementary cementitious ingredients. The recommended composition of UHPFRC, as outlined by Richard et al. [29], is shown in Table 1. In the second half of 2013, the French standards (AFGC) [30] for UHPFRC emerged as technical specifications and professional guidance often employed in UHPFRC design. Besides that, the German Committee of Reinforced Concrete [31] and Japan Society of Civil Engineers (JSCE) [32] guidelines in the design of UHPFRC have been commonly referred by investigators. These standards functioned as guidelines for suitable material procurement, formulation and modification of mixture design, and regulation of manufacturing techniques.

**Figure 1:**
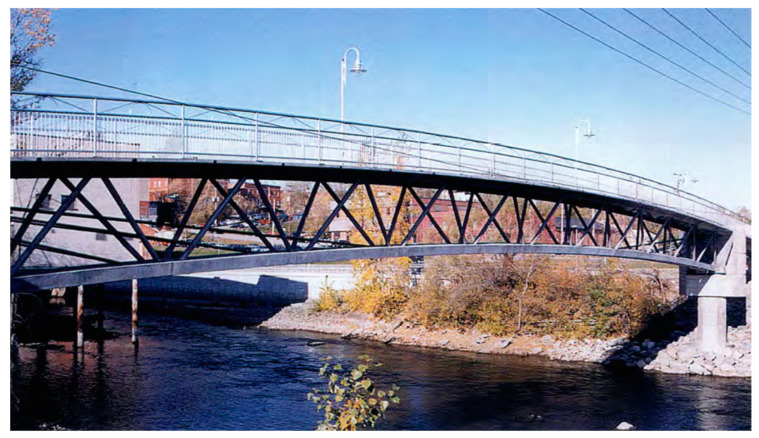
Sherbrooke pedestrian bridge in Canada [35,36].

**Figure 2:**
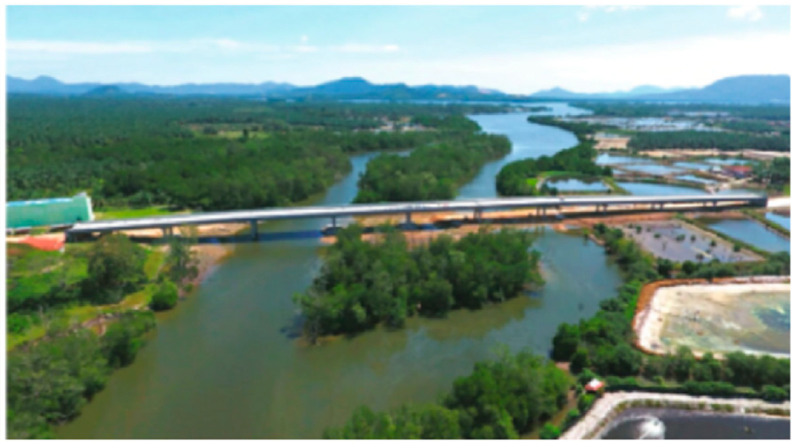
UHPFRC bridge in Perak, Malaysia [44,45].

The historical development of UHPFRC reflects decades of progress in concrete technology to accomplish outstanding durability, toughness, and flexibility. Prior to the 1980s, the initial advancement of high-strength concrete (HSC) commenced with attempts to diminish porosity and enhance packing density by vacuum mixing and thermal curing. These technologies attained compressive strengths of up to 510 MPa [29], although were constrained to laboratory settings due to their complexity and substantial energy demands. During the 1980s, innovations such as microscopic-defective free cement (MDF) and dense-silicate particulate (DSP) concrete were developed, with DSP attaining compressive strengths of up to 345 MPa and integrating steel fibers to mitigate brittleness concerns. Nevertheless, these materials encountered difficulties with workability and expense [33,34]. The 1990s spotted a pivotal advancement with the introduction of reactive-powder concrete (RPC), distinguished by its dense particle arrangement, higher binder concentration, and exceptional workability, allowing for compression strengths varying from 200 to 800 MPa [29]. RPC experienced the initial extensive uses of UHPFRC, exemplified by the building of the pedestrian walkway in Sherbrooke, Canada. [35,36], as illustrated in Figure 1. Since the 2000s, attention has turned to sustainable UHPFRC formulations that use supplemental cementitious materials like fly ash and slag, thereby diminishing environmental influence and production costs [37,38,39,40,41]. This period observed the increased global utilization of UHPFRC in bridges, facades, and roadways, reinforcing its significance as a revolutionary material for modern building [42,43]. Figure 2 depicts a completed UHPFRC bridge in Perak, Malaysia [44,45].

## 3. Durability of UHPFRC

### 3.1. Effects of Chemical and Environmental Aggression

The durability issues of reinforced-concrete members, including reinforcing corrosion, the reaction of alkali–silica, freeze–thaw cycles, and sulphate assault, are unavoidably engaged. This can expedite the degradation of RC members, hence reducing service life and elevating maintenance and strengthening/ rehabilitation costs [46,47,48]. UHPFRC denotes an extremely new combination of developed composite cementitious materials that exhibit significantly premium mechanical features and durability in comparison with conventional concrete [29]. UHPFRC is distinguished by significantly reduced porosity and a denser microstructure, ensuring its high permeability resistance relative to conventional concrete. Due to the high resistance of water permeability, UHPFRC prevents the infiltration of chemical compounds into concrete, especially chlorides, which eventually leads to the deterioration (corrosion) of steel-reinforcing bars [49,50,51,52,53,54,55]. The water absorption rate of UHPFRC was roughly 5 times less than conventional concrete, as reported in [56] and according to ASTM standards C127 [57]. Moreover, UHPFRC exhibits improved resistance to chloride permeability relative to traditional concrete, leading to superior corrosion resistance [58,59,60,61]. To assess the corrosion quantity reinforcing steel in high-performance concrete (HPC) and UHPFRC, Ghafari et al. [62] employed accelerated corrosion testing based on a standard test for corrosion potentials of uncoated reinforcement steel rebar in concrete (ASTM C876−15) [63]. The duration to crack in HPC was fifty percent less than that in UHPFRC. Fan et al. [64] evaluated the corrosion strength of implanted reinforcing rebars into UHPFRC. Moreover, the electro-chemical testing was performed on UHPFRC specimens sub-merged in a 3.5 weight % chloride of sodium liquid throughout the year. The findings indicated the absence of chloride, signifying no corrosion of the reinforcing steel rebar. On the other hand, freeze–thaw deterioration in concrete develops when molecules of water freeze and dilate beyond the material’s volume limitations. This causes distress in concrete, particularly when the induced pressure surpasses its tensile strength, ultimately leading to the expansion and collapse of fractures [65,66]. The exceptional freeze–thaw resistance of UHPFRC is attributed to its extremely impregnable matrix and decreased capillary pores [67,68]. Numerous investigations indicated that UHPFRC exhibited minimal degradation after 300–600 cycles of freeze–thaw, fulfilling an increased durability factor of more than 100 with very little to no loss of mass [58,61,69]. The tests were conducted based on the standards for resistance of concrete to immediate freezing and thawing (ACI 239R and ASTM C666/C666M) [70,71]. Pierard et al. [72] indicated that UHPFRC specimens achieving 140–160 MPa strengths exhibited no deterioration following 112 cycles. On the other hand, the overall mass losses of UHPFRC were 0.18, 0.50, and 0.62 following 500-1000-1500 cycles of freeze–thaw, respectively, as documented in [73].

### 3.2. Effects of Temperature and Fire

Fire resistance is the first of the characteristics which impact concrete construction durability. Concrete is a building material that has superior resistance to fire due to its low ability to conduct heat and substantial thermal capability. Increasing temperatures limit both the compressive and tensile strength of UHPFRC, leading to a reduced capability to withstand loads. This affects long-term durability as the material becomes increasingly vulnerable to mechanical and environmental stresses. Liu et al. [74] examined the fire efficacy and performance of powder concrete. The furnace temperature was heated according to the Japanese industrial standard JIS A 1304 [75]. The findings illustrated that the compression strengths of conventional concrete and UHPFRC loss were 41.5% and 37.8%, respectively, after 120 min of heating. On the other hand, the standard ASTM E119 [76] heating relationship curve was assumed for the fire examination to mimic actual conditions [77,78]. The compressive strength of UHPFRC specimens reduced between 30% and 50% over 1000 °C heating [77]. In addition, an increased steel fiber amount in the UHPFRC mix might mitigate strength loss by inhibiting crack propagation. The compressive strengths of UHPFRC specimens with steel fibers (1%, 2%, and 3%) and 200 °C heating improved by 24%, 4%, and 7%, and dropped when heating to 800 °C by about 82%, 78%, and 77%, respectively, as reported in [79]. Zheng et al. [80], explored the impact of temperature (ranging from 20 to 800 °C) on the compression and tension of reactive-powder concrete (RPC). The authors compared the experimental curves of compressive strengths-temperature with standard codes, namely EN. 1992-1-2, ACI 1989:216R–89R, and RakMK B4 1991 [81,82,83]. These standards codes are appropriate standards for normal concrete and high-strength concrete. The practical findings indicate that UHPFRC specimens containing 2% steel fibers avoided explosive spalling and markedly improved both tensile and compressive strengths. The compression strengths of models containing 1%, 2%, and 3% steel fiber at 800 °C are 39.7%, 43.0%, and 33.5%, respectively. Overall, the exceptional mechanical attributes and durability of UHPFRC render it an outstanding choice for structural systems, especially in difficult environmental conditions.

## 4. Evaluation Experimental Database

The number of different applications of UHPFRC is growing worldwide. Table 2 illustrates the concise summary of test results for strengthened RC structures in the literature. This section evaluates the experimental datasets in Table 2 to determine the arrangement of data concerning bonding technique, characteristics of the UHPFRC overlay, ultimate load, and failure mode. To date, 11 experimental works for strengthening RC members (slabs/beams) utilizing UHPFRC have been documented in the literature. The collected data were categorized for assessment according to the ultimate load and failure mode. Table 2 includes the overall characteristics of the RC slabs and beams, UHPFRC overlay properties, and the results of RC composite members.

### 4.1. Bonding Technique of UHPFRC

**Figure 3:**
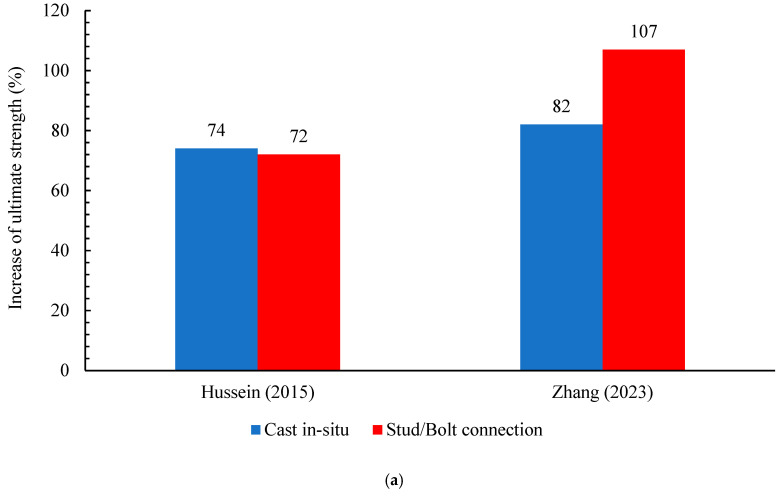

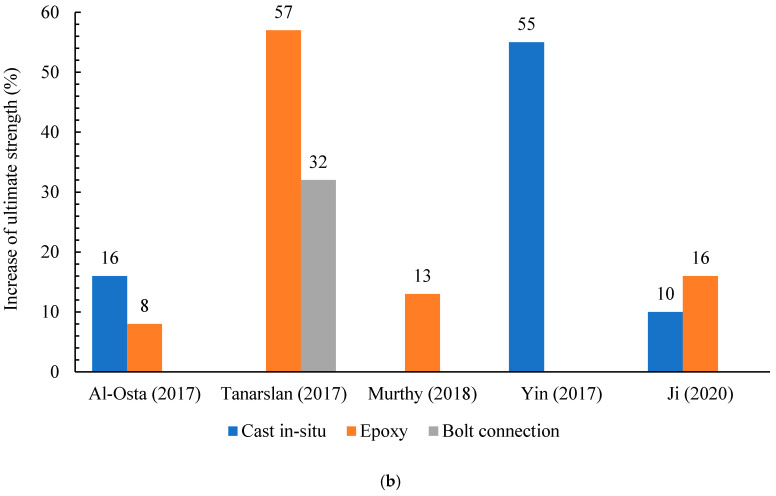
Percentages of ultimate strength improvement with various bonding techniques: (**a**) cast in-situ and stud connection bonding techniques; (**b**) cast in-situ, epoxy and bolt connection bonding techniques [84,85,86,87,91,92,94].

The effectiveness of the rehabilitation or strengthening method in enhancing the load-bearing capacity and durability of RC members relies mainly on the quality of the bond approach between the new layer of UHPFRC and the current RC concrete substrate. The resistance of the interface is determined by factors such as the kind of interface [95,96,97], the characteristics of the substrate (surface preparation) [98,99], the qualities of the strengthening material, and the use of a bonding method. In recent years, numerous strengthening techniques have emerged [100,101,102,103]. The maximum strength of reinforced RC members (slabs/beams) with an external layer of UHPFRC can significantly improve due to heightened mechanical features of the UHPFRC. Moreover, the bonding technique between the UHPFRC and the existing members is considered one of the essential aspects in increasing the stiffness and ultimate strength of retrofitted members. This provides multiple actions between the structural members and the UHPFRC layer, allowing them to work together and improving the effective ultimate strength. Therefore, in this part, the following bonding methods are discussed: (a) cast in situ, (b) dowel/stud connection, and (c) bonding agents (epoxy adhesive). To compare the efficacy of retrofitting by the UHPFRC layer in various publications, the increase in ultimate loads obtained by the experimental works was regularized by Equation (1). Figure 3 shows the increase in the ultimate strength of the RC members using different bonding techniques.

(1) $$Percentage\;increase\;in\;ultimate\;load\;P_{u}=\left(\frac{Pu}{Pu\;con}-1\right)\times 100$$

where (*Pu*) depicts the ultimate load and (*Pu con*) depicts the corresponding maximum load of un-strengthening members.

#### 4.1.1. Cast In Situ

**Figure 4:**
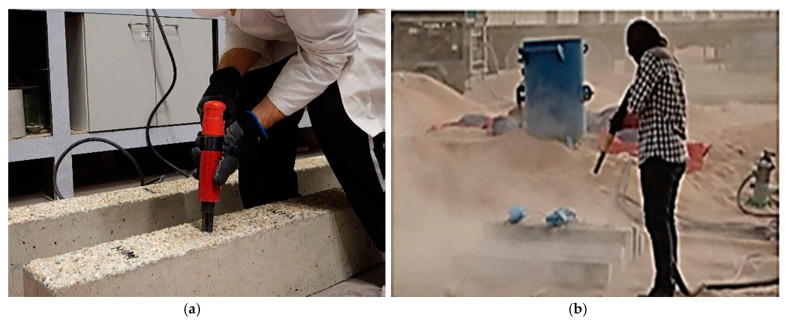
Interfacial preparation of RC beams: (**a**) pistol grip needle scaler process [89]; (**b**) sandblasting process [88].

Cast in situ signifies the process of pouring concrete directly into the RC structure (beam or slab) at the construction site. On the other hand, cast in situ includes various kinds of interaction facial practice: no preparation, rough preparation, and sandblasting. The procedure is commonly used for the non-preparation of the concrete substrate when the two layers are poured simultaneously [85]. As for the second type, rough preparation typically concerns creating a surface that enables a satisfactory bonding between the old and new concrete [104,105]. Various procedures were employed to achieve the necessary grade of roughness and various criteria were utilized to assess the roughness of concrete. In [106], the author utilized an air-chipping hammer to roughen the concrete surfaces, while in [89], a pistol grip needle scaler was used, as shown in Figure 4. The author of [107] used a water jet with high pressure for roughening the contact zone of the RC member. In [84], roughening was performed with a chisel and hammer. The faces of the RC beams were maintained moist for 10 min before wiping dry with a cloth. On the other hand, many researchers used a combination of roughening and epoxy adhesion [35,91,94,108,109,110]. The epoxy layer thickness in [91] was 5 mm. Sandblasting, or abrasive blasting, is a method employed to cleanse and create a rough finish on surfaces by using a machine that propels abrasive material (sand) at high velocity onto the concrete surface. This technique is commonly used for a range of purposes, such as preparing concrete surfaces before applying a strengthening layer. In [88], before pouring the UHPC layer, the faces of the RC specimens beam were roughened by using sand-blasting up to a depth of 2 mm, as demonstrated in Figure 4. Research demonstrates that roughening the surface of a concrete substrate can provide the desired binding. Figure 3 reveals that the enhancement in the ultimate strength of the strengthening specimens utilizing UHPFRC through the cast in situ technique ranged between 10% and 82%. Despite this improvement in ultimate strength, most specimens still suffer from the issue of premature debonding.

#### 4.1.2. Dowel/Stud Connection

**Figure 5:**
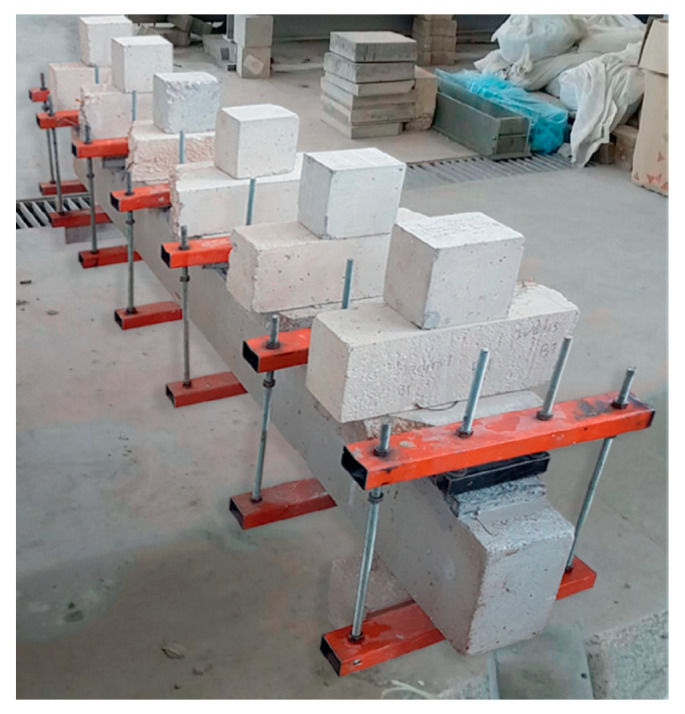
Application of prefabricated UHPFRC laminate bonding by bolt connection [94].

Dowel connections are frequently employed to guarantee robust adherence and structural coherence between an existing concrete surface and a fresh overlay or reinforcement layer. The process entails the insertion of steel dowels into the pre-existing concrete with a suitable bonding agent, such as epoxy, which extends into the new layer to establish mechanical interlock and facilitate load transfer. Anchorage length is essential for providing adequate load transfer between RC members and the UHPFRC layer when employing dowels or studs. According to the Building Code Requirements for Structural Concrete (ACI 318-19 section 17.6.4.1) [111], the embedded (anchorage) length of the dowels or studs in the RC concrete should be 2.5 times greater than the distance between the center of the dowels or studs to the edge of the RC concrete in the same direction. In addition, the minimum spacing between two anchors and the minimum distance between the anchor and the edge of RC members should be 6 times the diameter of the anchor. Inappropriate positioning or excessive spacing of dowels may result in stress concentrations, leading to cracking or failure at the connecting area. In [85], the author utilized two 10-M rebar as a dowels. These dowels were positioned at distances of 200 and 400 mm beside the steel support. They were employed as shear connectors, reinforcing the specimen’s beam. The experimental outcomes demonstrated that the embedding of dowels did not have a substantial influence on the shear ability of the RC composite beam. Zhang et al. [87] used the UHPC layer to fortify damaged RC beam specimens. After pre-damage, the beams’ bottom faces were cut to expose the coarse aggregate, which was then drilled with a 12 mm diameter and a 90 mm depth. Then, high-steel-strength bolts were installed, and holes of the steel bolts were cleaned and refilled with epoxy adhesive glue. The results showed that the reinforced beam substantially increased the resistance of cracks and maximum bearing capability. Besides that, the flexural ultimate load of beams that used the bolt connection technique demonstrated an increase of 25%, in comparison with the strengthened beam using the cast in situ bonding method, as portrayed in Figure 3. Tanarslan et al. [94] analyzed the flexural strengthening of reinforced concrete specimen beams utilizing prefabricated UHPFRC laminates by using a bolt connection method as well as an epoxy adhesive technique. Figure 5 illustrates the attached application of UHPFRC laminate [94]. From the experimental outcomes, as illustrated in Figure 3, the flexural ultimate load of beams that used the bolt connection and epoxy adhesive techniques exhibited increases of 32% and 57%, respectively, compared with the control beam. Moreover, in the BEAM-7 specimen at the same study, the author strengthened the beam with two prefabricated UHPFRC laminates: one in the tension face bonding by bolt connection and another one attached in the comparison side using the epoxy adhesive. The result of BEAM-7 showed that the maximum load improved by 208%, compared with a reference beam (BEAM-1). Despite the increase in the maximum load, the issue of early debonding between the existing structures and additional strengthening layers still dominates most studies.

#### 4.1.3. Bonding Agents (Epoxy Adhesive)

**Figure 6:**
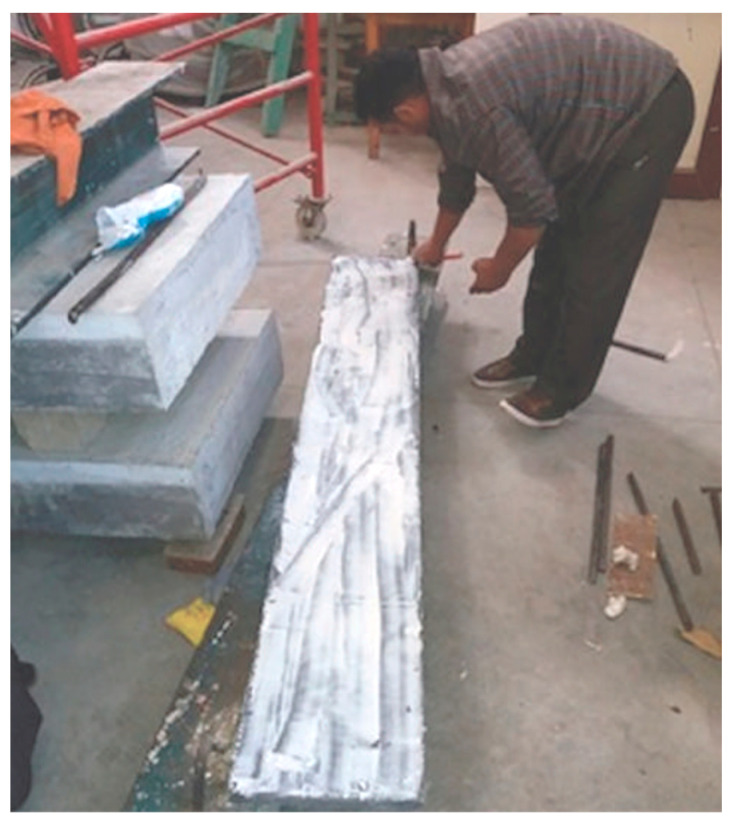
Coating the plate of UHPFRC with epoxy adhesive [90].

Bonding chemicals play a vital role in establishing a strong bond between preexisting concrete surfaces and new concrete overlays or strengthening layers. It improves the bonding between the two layers, allowing for the creation of a monolithic structure and improving the durability and load-bearing capability of the retrofitted area. Based on the ACI 503R-93 [112], the efficacy of epoxy application is significantly affected by the substrate surface, which must be robust, dry, and clean, absent from contaminants such as oil and dust, as well as the heat of the substrate to which the epoxy material is applied. The author used the UHPFRC plates for strengthening the RC beam with bonding agents [90]. Epoxy adhesive with two parts, namely the hardener and base, was employed. They were mixed entirely in a proportion of 1:1.5 utilizing a mixing instrument for 5 min until uniform in color, as illustrated in Figure 6. Experimental results demonstrate that the strengthening of RC beams (ST-2S) improved ultimate load capability and midspan deflection by 145% and 54%, respectively, in comparison with a benchmark specimen. M.A. Al-Osta et al. [86] analyzed the efficacy and efficiency of fortifying RC beams using the pre-fabricated UHPFRC strips bonding to the RC beams employing an epoxy layer. The results show that the UHPFRC jacket allowed for improvement in the cracking load with delayed crack propagation when compared with a reference specimen. Ji. et al. [92] studied the ultimate shear resistance of a strengthened RC beam with a UHPFRC layer. The results demonstrate that using the UHPFRC jacket and longitudinal reinforcing could effectively enhance the resistance of shear of the RC composite beams. From Figure 3, the ultimate load of beams that used the epoxy adhesive technique displayed an increase ranging from 8% and 57%, compared to the control specimens. According to previous studies in Table 2, despite the noticeable improvement in the peak loads of the specimens utilizing the epoxy bonding approach, the impact of cyclic loads on the performance of this method was not addressed.

### 4.2. Strengthening Configuration

**Figure 7:**
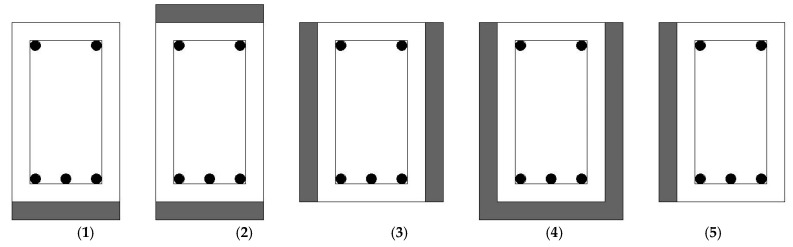
Usual patterns for strengthening using the UHPFRC layer: (**1**) T-sided (tension side); (**2**,**3**) 2-sided (two lateral sides); (**4**) 3-sided beam (three sides); (**5**) L-sided (one lateral side).

**Figure 8:**
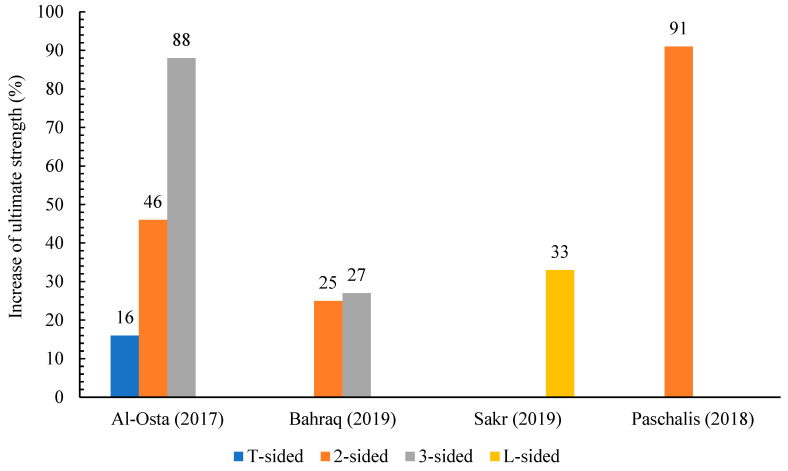
Improving the ultimate strength of experimental specimens with different strengthening configurations [86,88,89,90].

The literature review included four types of strengthening configurations: T-sided, 2-sided, 3-sided, and L-sided. The T-sided configuration involves strengthening elements on one side of the structural member (in compression or tension), generating a T-shape, and is commonly used to increase load-carrying ability and hardness. The 2-sided configuration has reinforcing features on two opposing sides of the structural member. The 3-sided arrangement includes strengthening components on three sides of the structural part, resulting in a more uniform augmentation. Finally, the L-sided design strengthens two neighboring sides of the structural element, resulting in an L-shape, which is utilized to increase strength and stiffness in two perpendicular directions. Figure 7 shows the different strengthening configurations. Combining [87,91], it is inferred that this strategy (T- sided configuration) is efficient with beams. The results indicate that the UHPFRC jacket improves the shear resistance of the RC composite specimens. In [90], the midspan deflection of the beam that used 2-sided configurations increased by 54% compared with the control beam. Beam specimens that were retrofitted on all three sides demonstrated a greater increase in capacity compared to specimens reinforced on only two sides [86]. By reinforcing one face of the RC beams [90], namely the L-sided beams, the efficacy of UHPFRC material in controlling the occurrence of diagonal shear cracks on the reinforced surface becomes evident. This is in contrast to the non-strengthened surface where such cracks are clearly visible. In contrast, the concrete behind the UHPFRC plate experienced cracking, resulting in the debonding of the UHPFRC. According to the data in Table 2, over 80% of the investigations focused on analyzing UHPFRC-RC composite (slabs/beams) under flexure, specifically with UHPFRC placed at the tension region. This is likely due to the fact that the UHPFRC displays superior tensile strength, ductility, and hardening strain behavior compared to traditional concrete. As a result, UHPFRC is better-suited for reinforcing or repairing structures on the side that experiences tension. Typically, the degradation or weakness observed in RC structures is caused by cracking resulting from the low resistance to stretching of normal-strength concrete (NSC). However, UHPFRC can overcome this inherent issue in NSC. Figure 8 shows that the increase in ultimate strength corresponds to the strengthening configuration.

### 4.3. Steel Fiber Ratio

**Figure 9:**
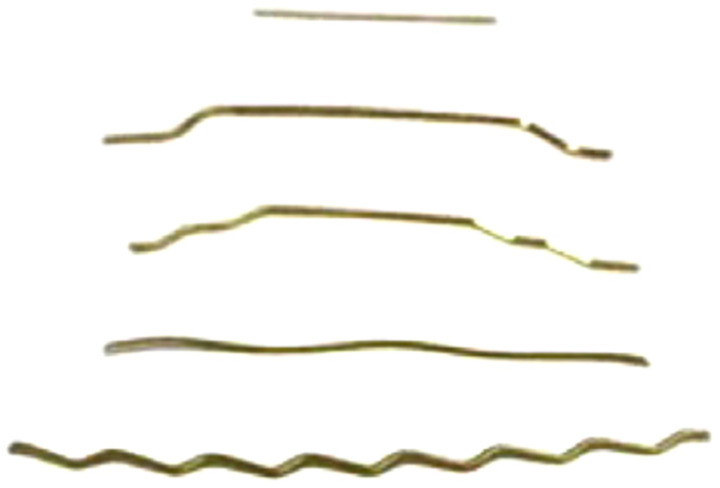
Typical classifications of steel fibers: micro straight, hooked, and corrugated fiber [134,135,136].

**Figure 10:**
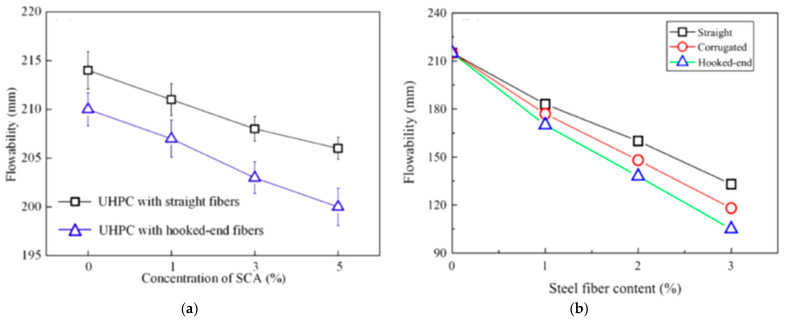
Flowability of UHPFRC using various sorts of steel fibers: (**a**) straight against hooked [131] and (**b**) straight against corrugated/ hooked [132].

**Figure 11:**
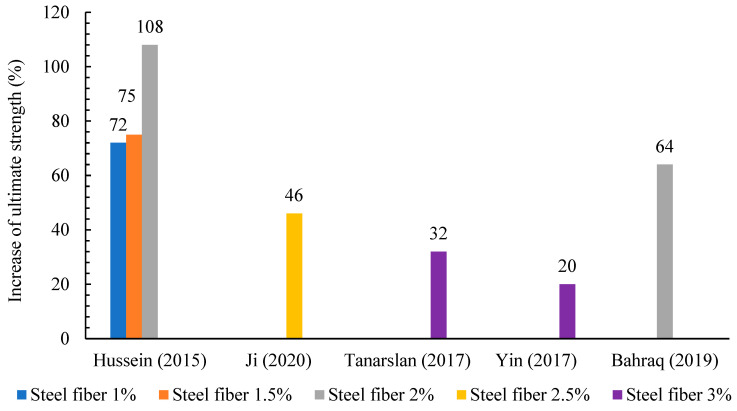
Impact of content steel fibers on the ultimate strength [84,85,88,92,94].

The mechanical characteristics of UHPFRC material, such as high strength and durability, depend on several factors. One of these factors is the presence of steel fiber [113] which bridges cracks and improves the tensile strength [114,115,116]. High steel fibers employed for concrete reinforcement have developed into several types, all designed to improve certain characteristics of a mix of concrete. Therefore, the sorts of steel fiber are summarized in Figure 9. As seen in Figure 9, three common types of steel fiber were reviewed: the micro-straight smooth fiber, hooked-end fibers with various hooks at the ends, and corrugated fibers. On the other hand, the researchers conducted many investigations to determine the ideal ratio of steel fibers in the UHPC mix. [117,118,119]. The fibers volume ratio added to UHPC ranges from 1.5% to 3%, as shown in Table 2; nevertheless, the economical fiber ratio was proposed to be 2% by volume. The first version of UHPFRC had 13 mm length micro-straight steel fibers [29]. Hussein et al. [85] used straight steel fibers with a length and diameter of 13 and 0.2 mm, respectively, with above 2500 MPa tensile strength, and incorporated three different steel fiber contents (1%, 1.5%, and 2%) in the UHPC layer. The findings in [85] demonstrated that using a 2% fibers volume content in the UHPC mix significantly improved the flexural ability of RC specimen’s beam by 108% with high ductility. Tanarslan [94] examined the flexural response of strengthened beams utilizing prefabricated UHPFRC laminates, for which 3% volume micro-steel fibers with a 13 mm length and 0.2 mm diameter were employed in the UHPFRC mix. The failure [94] resulted from the concentration of cracks and subsequently the delamination of the UHPFRC laminate reduced the influence of the laminate to the maximum load capacity as well as to the ductility performance. Another researcher [120] employed 0.2 mm diameter straight steel fibers with 19.5 mm and 16.3 mm lengths in the UHPC mix. Ryu et al. [115] examined the response of the UHPFRC that contained mixed straight fibers of various lengths (13, 16.3, and 19.5) mm at the contents of 1.5 and 2%. The utilization of mixed steel fibers [115] demonstrated notable enhancement in stiffness and flexural strength in comparison to employing a single kind of steel fibers. In contrast, numerous studies [121,122,123,124,125,126,127,128] have endeavored to employ a hooked class of steel fibers for a new reinforcing in the UHPFRC mixing. K. Wille et al. [129] studied and enhanced the fracture energy of UHPFRC employing relatively lower fiber contents. They [129] utilized a 2% hooked-end fiber content volume with a 30 mm length and 0.38 mm diameter in the UHPC mix. The UHPFRC with the hooked steel fibers [129] displayed good strain capacity, tensile strength, and rupture energy. The impact of corrugated steel fibers on the fracture energy and properties of the UHPFRC material was investigated by Soloviev et al. [130]. The incorporation of 2% brass-coated corrugated steel fiber [130] with diameter and length of 0.3 and 15 mm, respectively, slightly improved the compressive strength. Liu et al. and Wu et al. [131,132] found that the UHPFRC using hooks and corrugated steel fibers indicated lower flowability than that straight, as portrayed in Figure 10. This was primarily forced by the propensity of the deformed steel fibers to entangle with one another, resulting in an increase in the friction energy between components of the UHPFRC paste compared to straight steel fibers [133]. Figure 11 illustrates the improvement in ultimate strength of concrete members reinforced by UHPFRC with different content volumes. At 2% steel fiber content, the ultimate strength showed the highest improvement and increased by 108%, indicating its optimal performance [85], as shown in Figure 11. Besides that, the lowest percentage of improvement in the maximum load was 20% when using 3% steel fiber [84]. These results indicate that, while adding steel fibers enhances strength, 2% emerges to be the most effective content, as both higher and lower percentages exhibit decreasing returns.

### 4.4. UHPFRC Thickness

**Figure 12:**
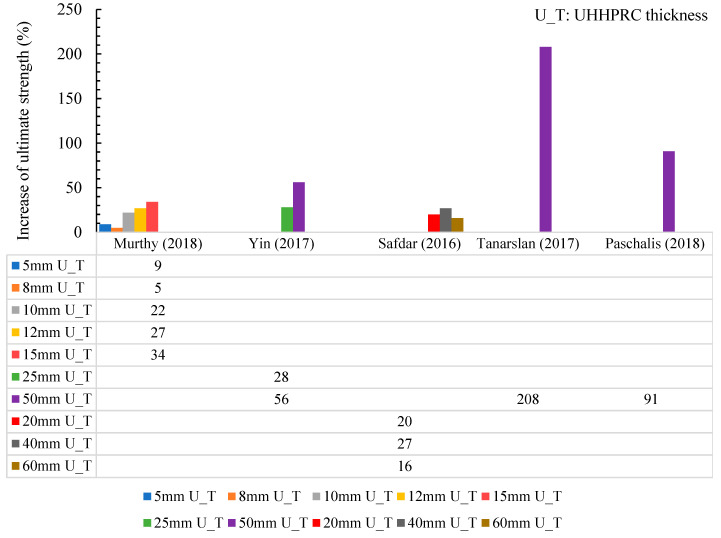
Influence of UHPFRC thickness on the ultimate strength [84,89,91,93,94].

The thickness layer of UHPFRC employed for reinforced structural members (beams or slabs) could vary depending on the design requirements, the level of structural enhancement required, and the specific project conditions. Commonly, UHPFRC layers range from 20 mm to 50 mm for strengthening purposes. The thickness of UHPFRC used in strengthening reinforced concrete structures such as beams or slabs is an essential factor that balances structural enhancement and material efficiency. UHPFRC, known for its superior strength, ductility, and durability, allows for relatively thin layers to achieve effective reinforcement. According to the existing experimental investigation results, as outlined in Table 2, the thickness of UHPFRC ranged from 5 to 150 mm. The application of 5 mm UHPFRC thickness was developed in beam-RB2 [91]. Beam-RB2 was reinforced with pasting UHPC strips with epoxy adhesive. The peak load of the retrofitted beam (RB2) increased by 9.38%, compared with benchmark beam (CB1). Otherwise, Hussein et al. [85] used the strengthening layer with 150 mm thickness in different bonding techniques. The flexural load improved in the range of 60–108% for all strengthening methods. The highest increase was reported by Yin. et. [84] when using a 50 mm thickness UHPC to reinforce the slab. Furthermore, in [84], a 50 mm thick UHPFRC jacket was recommended for employment due to the softening behavior that appeared in thicker UHPFRC. This was explained in [84], where as the UHPFRC jacket thickness improved, UHPFRC tensile strength was reduced because more irregular fibers were almost observed in the UHPFRC’s upper face, and the strength lowering was more noticeable in the thicker UHPFRC. Thus, the optimal UHPFRC thickness might be 50 mm, according to the existing data. Figure 12 shows that the increase in ultimate strength corresponds to UHPFRC thickness.

### 4.5. Reinforced UHPFRC Layer

**Figure 13:**
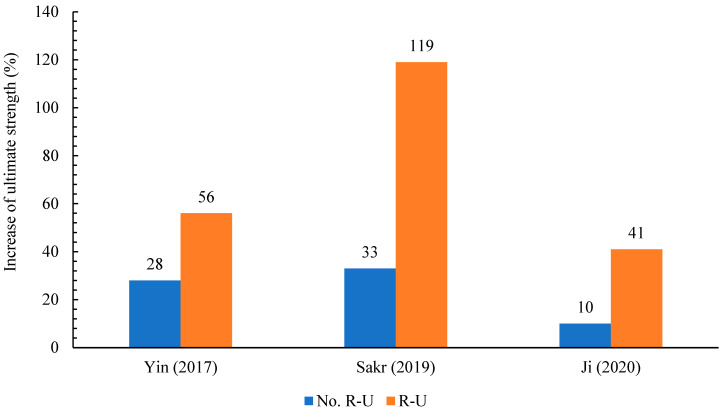
Impact of reinforcement steel in the UHPFRC on the ultimate strength [84,90,92].

Adding steel-reinforcement bars into the UHPFRC was effective in improving the hardness and ultimate load of the fortified beams and slabs. Besides that, the embedded steel bars substantially enhance the capability of UHPFRC for regulating the ultimate width of cracks, by increasing the tensile strain [137,138,139,140,141,142]. The reinforcement rebars absorb the tensile stress from macro cracks until the rebar steel yields and fails, thus improving both the capability and flexibility of the RC part. However, as seen in Table 2, most investigations reinforced the UHPFRC with steel rebars. Figure 13 demonstrates the impact of reinforcing rebars in the UHPFRC layer on the RC member capacity. For the reinforced UHPFRC layer, the reinforcement ratio was mainly especially lower than the reinforcement ratio in existing reinforced concrete members. This is due to the cross-sectional area of the UHPFRC being less than that of the RC members (beams/slabs). In addition, according to the Guide of Concrete Repair ACI 546 R-96 Section 4.1.11 [143] and ACI 224.1R-93 [144], the minimum thickness of concrete overlay layers varies from 38 mm to any suitable thickness, relying on strengthening or repairing requirements of the specific case. On the other hand, due to a lack of design standards codes for the minimum thickness of UHPFRC layer that is used for strengthening RC members, most researchers recommended the thickness range of 20–50 mm, also depending on the strengthening requirements. However, Table 2 indicates that the reinforced UHPFRC layer alters the failure modes observed in unreinforced UHPFRC. This occurred in [94], as in the absence of reinforcement steel, the UHPFRC jacket developed a localized macrocrack that resulted in complete failure, irrespective of interface therapies. On the other hand, when steel reinforcement was incorporated into the UHPFRC layer, the concrete experienced crushing due to mechanical anchors, while the UHPFRC jacket exhibited delamination from the epoxy. In [90], the authors examined the shear response of reinforced RC beams with/without steel rebars in the prefabricated UHPFRC jacket. The steel-reinforced UHPFRC layer exhibited a more densely distributed pattern of macrocracks and pronounced strain-hardening performance, ultimately failing in flexure. Also, Yin. et al. [84] verified that the use of reinforcing rebar in the UHPFRC layer resulted in a noteworthy improvement in ultimate strength. Furthermore, the diameter size of steel rebar utilized in the UHPFRC overlay was investigated. The UHPFRC layer overlay in [93] included two steel-reinforcing rebars with a 16 mm diameter. Two steel bars with a 10 mm diameter were employed in the UHPFRC layer [89]. The UHPFRC layer in [94] contained three steel bars 8 mm in diameter. According to this review, reinforcement steel bars and a smaller-size diameter were typically more employed in the UHPFRC layer overlay than in the steel reinforcement in the existing beams or slabs. The experimental results of the specimens (slabs/beams), as depicted in Figure 13, indicate that reinforcing the UHPFRC layer with rebar steel enhances the maximum strength of the specimens. Although there are many benefits to reinforcing the UHPFRC layer, it entails specific drawbacks. One key issue is that, although UHPFRC offers some protection, the potential for steel reinforcement corrosion remains a concern, particularly if cracks develop or bonding issues occur. In addition, incorporating steel reinforcement increases the overall weight of the RC structure member, potentially affecting the strengthening system. This might threaten the long-term effectiveness of the strengthening system, particularly under severe environmental conditions.

### 4.6. Failure Mode

**Figure 14:**
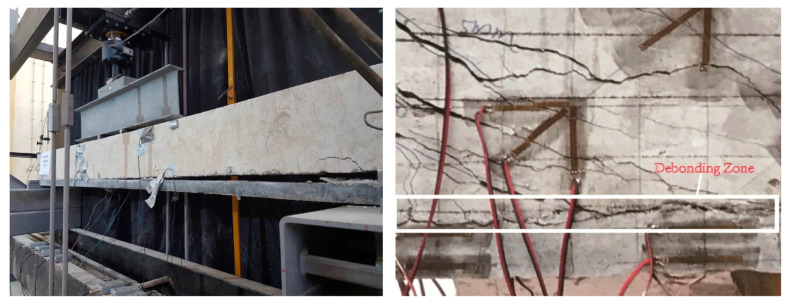
Debonding failure at the interface between UHPFRC and NSC [92,94].

The failure modes of RC elements (slabs/beams) strengthened by an exterior layer of UHPFRC are complex and diverse. Comprehending these modes is crucial for the design and assessment of reinforced structures. Therefore, four main types of failure modes, namely flexure (*F*), shear (*S*), peeling (*P*), and de-bonding between UHPFRC and NSC (*D*) were reported. The peeling failure (*P*) mode of the UHPFRC–NC interface was observed in [87]. This special case of peeling of the UHPFRC–NC interface happened due to the mechanically anchored UHPFRC jacket without implanted reinforcement steel bars. Upon the occurrence of this failure, the slips between the UHPFRC–NC interface and steel plate–UHPFRC interface developed, followed by the growth of the interaction horizontal and diagonal cracks in NC. For the flexure (*F*) and shear (*S*) failure modes, they occurred in almost all of the studies reviewed in Table 2 because these experimental specimens were reinforced with UHPFRC on the tensile zone, leading to the crushing of the NC substrate in the compression face. Additionally, numerous closely spaced cracks formed in the UHPFRC layers due to the strain-hardening features. On the other hand, there were dual types of failure that occurred in some experimental studies, as in [84,86,87,88,90,93,94]. In [91], the flexural failure modes of fortified beams were similar to control RC beams that comprise the yielding of rebar steel and are followed by the crushing of NSC. In contrast, in [94], all composite beams from series 1 and 2 experienced shear failure as a result of principal tension stress within the NC or UHPFRC layers. The tensile force in the UHPFRC layers surpassed the concrete’s tensile stress, causing a diagonally tensile crack oriented perpendicular to the orientation of the primary tensile stress. On the other hand, in most failure cases, the de-bonding failure of the contact between the UHPFRC and NC is an essential factor, as it directly affects the composite action of the strengthening system. Debonding failure in strengthening approaches utilizing UHPFRC can appear in several forms, commonly related to interface characteristics, bonding strategies, and applied loads. The debonding failure mode (*D*) between UHPFRC and NSC layers was observed in [84,86,87,89,93,94]. In [84,89], the specimens with unreinforced UHPFRC layer failed in debonding between the UHPFRC and the NC layers at the composite interface. It is essential to note that, in [90], the mechanism of debonding failure resulted from the fracture of the NC underneath the UHPFRC plate. Tanarslan [94] investigated the strengthening of flexural beams utilizing pre-fabricated UHPFRC laminates with two distinct connection methods: bolted connections and epoxy adhesion. The results demonstrated that both bonding techniques experienced premature debonding before reaching the maximum load, as depicted in Figure 14. The maximum shear resistance of RC composite specimens was investigated by Ji et al. [92]. From Figure 14, the presence of higher shear stress with several fine cracks on the NSC adjacent to the interfacial region with UHPFRC led to the softening of the concrete in that area, leading to the development of a debonding zone.

The efficacy of any strengthening strategy of structural elements depends mainly on the bonding techniques between them. Early deboning can appear on various sides of the system. Nevertheless, with external strengthening methods, debonding might occur at the NSC–UHPRFC interface, between the NSC and the adhesive layer, between the adhesive layer and the UHPFRC, and adjacent to the failure in the adhesive materials. A variety of parameters influence bonding system performance and must be analyzed and assessed in every new bonding configuration.

### 4.7. Mechanical Performance of UHPFRC-Reinforced Concrete Members

To comprehensively evaluate the effectiveness of UHPFRC in strengthening concrete structures, this section examines and summarizes its mechanical performance in reinforced-concrete (RC) elements subjected to extreme loading conditions, including impact, explosion, and cyclic loading. Chun et al. [145] investigated the impact resistance of RC beams employing different types of UHPFRC. Ten large-scale RC beams were manufactured and subjected to testing under static and impact loading cases. The outcomes indicated that the UHPFRC beam achieved the highest peak reaction load upon impact, roughly 39.4% more than that of the NC beam. On the other hand, the improvement in impact resistance in RC beams utilizing UHPFRC with three varieties of steel fibers and two fiber volume portions (0.75–1.5%) was conducted by Yoo et al. [146]. The reinforced UHPFRC beams exhibited a strength enhancement of around 6% with a fiber volume ratio of 1.5% in comparison to the benchmark RC beam. Li et al. [147] investigated the performance of retrofitted reinforced concrete beams with different strengthening configurations of UHPFRC underneath both individual and repeating explosion loads. In all strengthened beams with different configurations, the results show decreased deflections (ranging from 20% to 60%) when compared to the control beam. Nevertheless, the recurrent blasts eventually resulted in the localization cracks and rupture of steel rebars. Nine fortified composite beams employing UHPFRC were subjected to cyclic loading tests conducted by Wang et al. [148]. The test parameters comprised UHPFRC thicknesses (200–360 mm), reinforcing proportions, and load levels. The findings from the testing of composite beams indicated enhanced fatigue lifetimes by 4.2–7.0 times for those employing 200–360 mm thick UHPFRC, relative to the reference beam. Liu et al. [149] conducted an experimental assessment of the shear performance of reinforced concrete beams fortified with a U-shaped UHPFRC jacket under both static and fatigue load conditions. Five beams with varying characteristics, including UHPFRC thickness and the shear span-depth ratio of the beam (a/d), were subjected to static loads to ascertain ultimate shear strengths, subsequently followed by fatigue testing on similar beams at 30 to 70% of maximum shear strengths at a frequency of 4 Hz. The UHPFRC jacket enhanced the fatigue lifespan of reinforced concrete beams. In composite beams with 2.0 and 3.5 a/d ratios, the fatigue life increased from 75 to 951 cycles and from 12,525 to 48,786 cycles, respectively. The mechanical performance of UHPFRC-reinforced concrete elements under severe loading conditions exhibits notable enhancements in impact resistance, explosion mitigation, and fatigue durability. Research indicates that UHPFRC improves peak impact load capacity, reduces deflections under blast loads, and prolongs the fatigue life of reinforced concrete components. These findings highlight the exceptional strength, durability, and resilience of UHPFRC, enabling it an exceptionally effective material for strengthening and retrofitting structures exposed to severe loading environments.

## 5. Numerical Studies for Strengthening Systems

Numerical analyses in structural engineering, especially in the context of strengthening structural members using UHPFRC, generally apply to simulate the responses of these members under various loading and boundary conditions. Finite element (FE) analyses employing commercial software are an outstanding approach to assess the structural performance and impacts of the strengthening method. FE analysis allows for the simulation of various scenarios, such as stress distribution, deflection, and crack patterns under different load conditions, offering insights into responses of beams and slabs retrofitted with the UHPFRC layer. Finite element studies were performed in this literature review to enhance the understanding of the performance of composite structural members reinforced by UHPFRC. The comparison between experimental works and numerical results in terms of ultimate load was summarized in Table 3. Numerous FE investigations used commercial software like ATENA, ABAQUS, DIANA 9, and LS-DYNA [86,88,89,90,91,93,150]. Notably, ATENA software provides a specialized material model for fiber-reinforced concrete (FRC) that complies with the fib Model Code 2010 [151]. This guarantees that the numerical simulation precisely reflects the strain-hardening and strain-softening characteristics of UHPFRC, resulting in more dependable [2,3] renditions of structural performance Two essential aspects of FE models were examined in this literature review, which might impact and specify the accuracy of the members’ response. First, the constitutive model of the UHPFRC layer under compression and tension conditions, and second, the interaction contact response between the NC–UHPFRC layers.

### 5.1. Tensile and Compressive Constitutive Relationships of UHPFRC

**Figure 15:**
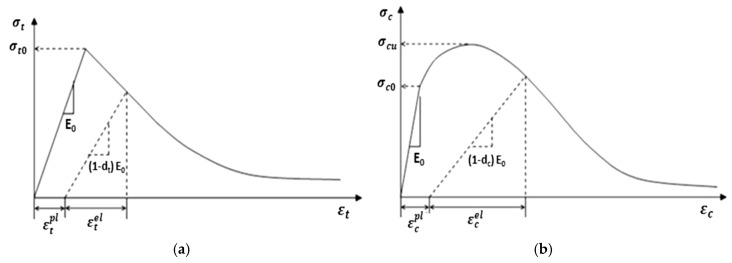
Constitutive behavior of concrete [88]. (**a**) Tension (**b**) Compression.

UHPFRC has distinct tensile and compressive constitutive behaviors that markedly contrast with those of conventional concrete, attributable to its higher strength, strain-hardening properties, and enhanced ductility. Comprehending these relationships is crucial for precise modeling of UHPFRC in structural applications. Generally, the concrete damages plasticity model (CDPM) was utilized for simulation of nonlinear constitutive response on both sides of the compression and tension of the UHPFRC composite element [86,88,91], as depicted in Figure 15 [88]. The CDP model of the UHPFRC layer from the tensile response should include strain-hardening and softening phases [152], as illustrated in Figure 15. This relationship encompasses the initial linear elasticity, succeeded by strain hardening, and then softening beyond the peak stress. In contrast to conventional concrete, UHPFRC retains load-bearing capacity post-peak stress owing to fiber-bridging effects. Most researchers used Equations (2) and (3) to calculate the compression and tensile constitutive law of UHPFRC. Besides that, UHPFRC stress–strain relationships should be converted to stress-inelastic strain using Equations (4) and (5) to determine the inelastic strain. Lastly, Equations (6) and (7) were employed to calculate the damage parameters of concrete in the compressive and tensile strengths for the UHPFRC.

The compression and tensile stress–strain of UHPFRC are calculated as the equations below:

(2) $$\sigma_{c,c}=\left\{\begin{array}{c} f_{c}\frac{n\xi -\xi^{2}}{1+\left(n-2\right)\xi }\;0<\varepsilon \leq \varepsilon_{0}\\ \;\\ f_{c}\;\varepsilon_{0}\leq \varepsilon_{c,c}\leq \varepsilon_{cu} \end{array}\right.n=\frac{Ec}{Es};\xi =\frac{\varepsilon_{cu}}{\varepsilon_{0}}$$

(3) $$\sigma_{c,t}=\left\{\begin{array}{c} f_{t}\;\frac{\varepsilon_{t}}{\varepsilon_{t0}}\;0<\varepsilon_{c,t}\leq \varepsilon_{t0}\\ \;\\ f_{t}\;\varepsilon_{t0}<\varepsilon_{c,t}\leq \varepsilon_{tu} \end{array}\right.\varepsilon_{t0}=\frac{f_{t}}{Ec};\varepsilon_{tu}=\frac{{30f}_{t}}{Ec}$$

Consequently, the stress-inelastic strain relationship can be determined using the following equations:

(4) $$\varepsilon_{c}^{in}=\varepsilon_{c}-\sigma_{c}/E_{0}$$

(5) $$\varepsilon_{t}^{in}=\varepsilon_{t}-\sigma_{t}/E_{0}$$

Lastly, the damage parameters of concrete in compressive and tensile strengths are calculated using the equations provided below:

(6) $$D_{t}=1-\frac{\sigma_{t}E_{c}^{-1}}{\varepsilon_{t}^{pl}\left(\frac{1}{b_{t}}-1\right)+\sigma_{t}E_{c}^{-1}}\varepsilon_{t}^{pl}=b_{t}\varepsilon_{t}^{in}$$

(7) $$D_{c}=1-\frac{\sigma_{c}E_{c}^{-1}}{\varepsilon_{c}^{pl}\left(\frac{1}{b_{c}}-1\right)+\sigma_{c}E_{c}^{-1}}\varepsilon_{c}^{pl}=b_{c}\varepsilon_{c}^{in}$$

$E_{c}$ indicates the elastic-modulus, $f_{c}$ and $f_{t}$ denote the UHPFRC strength )compressive and tensile(, Es denotes the equal secant modulus at maximum state, $\varepsilon_{cu}$ and $\varepsilon_{tu}$ denote the ultimate compression and tension strain of UHPFRC, $\varepsilon_{0}$ and $\varepsilon_{t0}$ denote the peak compression tension and strain, $D_{t}$ and $D_{c}$ indicate the parameters of damages in tension and compressive, respectively, $\sigma_{c}$ and $\sigma_{t}$ indicate the stress in compressive and tension, $\varepsilon_{t}^{pl}$ and $\varepsilon_{c}^{pl}$ denote the plastic strain linked with the tensile and compression stress, respectively, $b_{c}and$ $b_{t}$ are invariant values ranging between $0<b_{c}\;and\;b_{t}<1.$

### 5.2. Interaction Contact Response Between the NC–UHPFRC Layers

**Figure 16:**
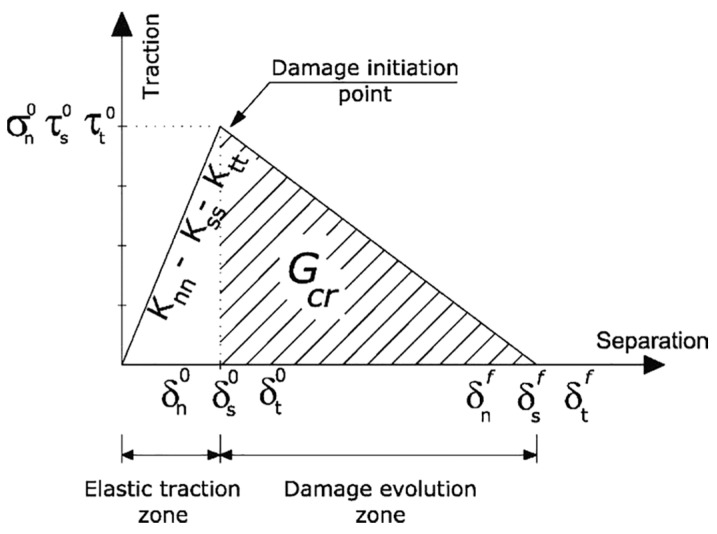
Bilinear traction separation law in the nearest interface area [152].

**Figure 17:**
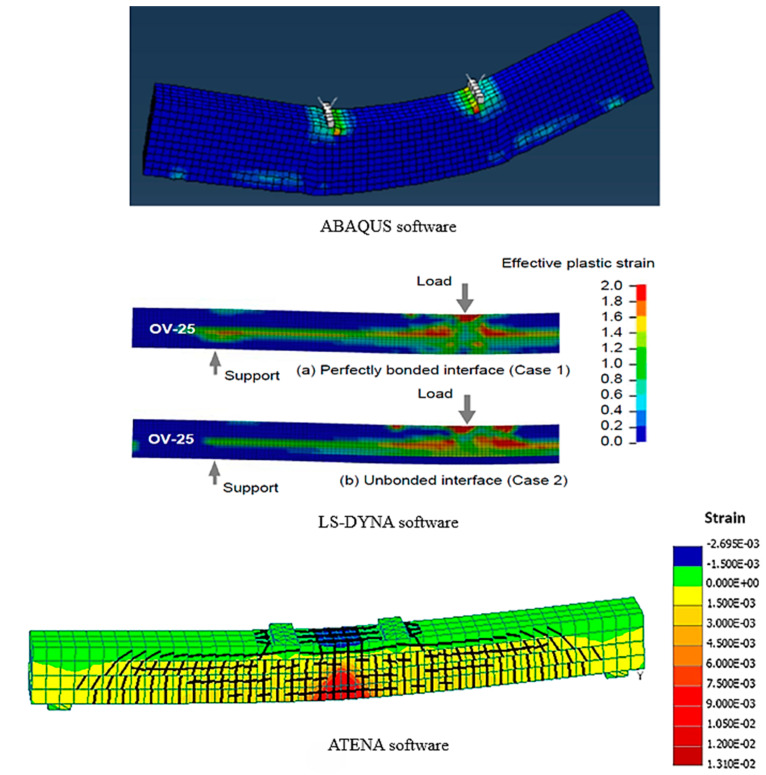
Comparison of cracking patterns of FEM models in different calculation software.

To appropriately predict the behavior of UHPFRC–NSC composite members, besides modeling of the material, attention must be paid to the interaction contact modeling between UHPFRC and RC elements. Numerous researchers assumed the perfect bond (Tie contact) [86,91,93,108,153] between UHPFRC–NSC layers to withstand the debonding issue based on the experimental observance. On the other hand, some researchers [89,90,150,152] initiated the consideration of interaction modeling on composite elements rather than the perfect bond assumption. The cohesive element zone model was used in the simulation to describe the interaction contact between UHPFRC–NSC layers which authorizes slip and debonding to occur such as in the experimental test [80,131]. Three main parts in the cohesive model are linear elastic traction separation, damages initiation, and the damages evolution, as shown in Figure 16 [152]. Paschalis. et al. [89] represented the interface contact by 2D interaction contact elements and by utilizing a friction coefficient, beside a cohesion of 1 and 1.8 MPa, respectively. Besides these interface models, an equivalent elements approach to simulate the interaction bond for UHPFRC–NSC layers was suggested [150]. The numerical findings utilizing the equivalent element technique demonstrated a good relationship with experimental results in contrast to simulation outcomes employing a perfect bond or a fully unbonded interface. Figure 17 shows a comparison of the cracking and destruction of FEM models in various calculation software [89,91,150]. Additionally, Zhu et al. [154] employed two different interaction modeling strategies to simulate the damaged RC slabs fortified with the UHPFRC layer: friction alone based on ACI [155] and adhesion with friction according to AASHTO [156]. The outcomes indicate that the finite element modeling utilizing AASHTO principles is superior in predicting load–deflection curves compared to the ACI concept. The strength bond of the UHPFRC–NSC under shear test, flexural, torsional loading, and punching shear test was comprehensively investigated [157,158,159,160,161,162,163,164,165,166]. Nevertheless, there is insufficient knowledge regarding the behavior of strengthened composite slabs under cyclic loads. In the strengthening solutions involving UHPFRC, a critical factor of the composite elements is ensuring the interaction bond strength exceeds the tensile stress of the RC member. Given that those interface parameters influence both the capacity and failure mode of composite structures, it is essential to develop an improved FE model utilizing appropriate interface modeling that can effectively estimate the response of the RC composite members, evaluate debonding hazards, and optimize the strengthening techniques using UHPFRC.

## 6. Analytical Models for Strengthening Systems

**Figure 18:**
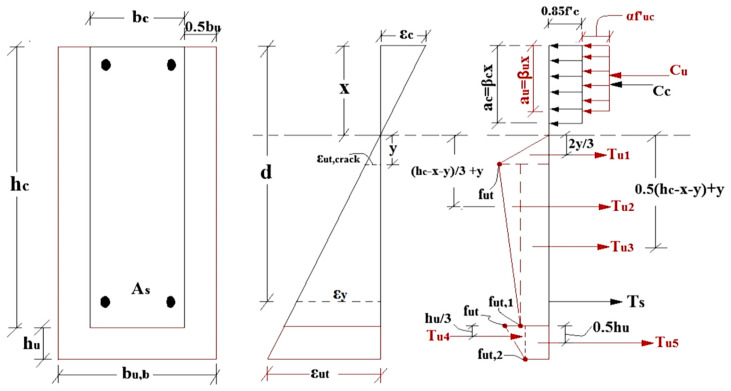
Analytical model scheme.

The major purpose of analytical models is to present formal computational equations and relationships in a simplified manner, allowing designers, engineers, and manufacturers to compute and devise appropriate methods, materials, and configurations for each strengthening scenario. The analytical investigation of various RC structural approaches by formulas and calculations has been successful in recent decades. The domains of retrofitting and strengthening are closely associated with various scientific disciplines, including the analysis of structures, composite building materials, and the science of adhesion bonding materials. A number of countries have commenced the development and implementation of their own rules, standards, and design guidelines. Despite being relatively recent in comparison to RC structures, these guidelines have evolved swiftly and have substantially impacted the standardization of several strengthening procedures. The analytical models of RC members were primarily derived from reinforced concrete design. Two primary assumptions for analytical models were typically employed: the Bernoulli hypothesis (plane parts remain planar) and monolithic behavior (no sliding transpires at the interface between UHPFRC–NSC) [167]. The expected moments for initial and ultimate cracks derived from the suggested analytical approaches for flexural enhancement align more closely with the practical test outcomes. In [86], the researcher used the analytical/mechanistic models of their cross-section to estimate the moment capability of the fortified beam with UHPFRC. To predict internal forces, the analysis model of the beam employed internal stresses according to the stress and strain distribution section, as given in Equations (8)–(15). Besides that, to calculate the failure moment in a three-sided reinforced configuration, the authors of [86] employed an equivalenting rectangular block stress for UHPFRC under compression according to [168], whereas bilinear stress and strain curves were utilized for UHPFRC under tension. Figure 18 shows the scheme of the analytical model [86].

Calculate the compressive and tension resultant forces in the sections NSC and UHPFRC as below.

(8) $$C_{c}=0.85f_{c}^{\prime }\beta_{c}xb_{c}$$

(9) $$C_{u}=\alpha_{u}f_{uc}^{\prime }\beta_{u}xb_{u}$$

(10) $$T_{s}=A_{s}f_{y}$$

(11) $$T_{u1}=0.5f_{ut}b_{u}y$$

(12) $$T_{u2}=0.5\left(f_{ut}-f_{ut,1}\right)\cdot b_{u}\left(h_{c}-x-y\right)$$

(13) $$T_{u3}=b_{u}\left(h_{u}-x-y\right)f_{ut,1}$$

(14) $$T_{u4}=0.5h_{u}\left(f_{ut}-f_{ut,k}\right)b_{u,b}$$

(15) $$T_{u5}=h_{u}b_{u,b}f_{ut,2}$$

The value of *x* is acquired by the equilibrium of forces reached, as in Equation (16) below.

(16) $$\Sigma F=\left(C_{c}+C_{u}\right)-\left(T_{s}+T_{u1}+T_{u2}+T_{u3}+T_{u4}+T_{u5}\right)=0$$

The predicted moment capability is calculated by taking the moment of internal forces regarding the neutral axis location, as presented in Equation (17) below.

(17) $$\begin{array}{cc} M_{\mathrm{pred}\;}= & C_{c}\left(x-\frac{a_{c}}{2}\right)+C_{u}\left(x-\frac{a_{u}}{2}\right)+T_{s}(d-x)+T_{u1}\left(\frac{2y}{3}\right)\\ & +T_{u2}\left[\frac{1}{3}\left(h_{c}-x-y\right)+y\right]+T_{u3}\left[\frac{1}{2}\left(h_{c}-x-y\right)+y\right]\\ & +T_{u4}\left(h_{c}+\frac{1}{3}h_{u}\right)+T_{u5}\left(h_{c}+\frac{1}{2}h_{u}\right) \end{array}$$

*Cc* and *Cu* represent the compressive resultant forces in NSC and UHPFRC, respectively; *Ts* represents tensile resultant forces in reinforcing steel; *Tu1*, *Tu2*, *Tu3*, *Tu4*, and *Tu5*, represent tensile resultant forces in UHPFRC; $f_{c}\;and\;f_{ut}\;$denote compressive strength of NSC and UHPFRC, respectively.

**Figure 19:**
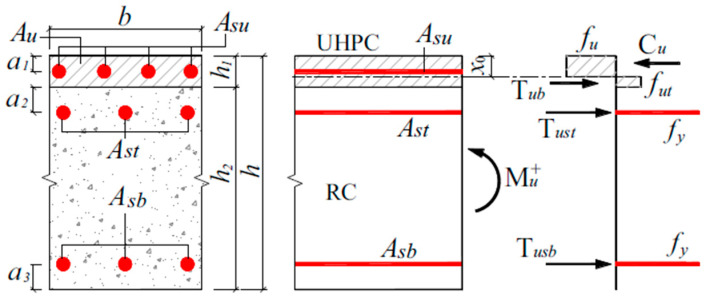
Layout of an analytical model of fortified slab.

Zhang et al. [169] conducted a comparative analysis of analytical models and experimental studies regarding the flexural capability of slabs reinforced with UHPFRC. The analytical calculation was developed according to the mode of failure, identifiable through the corresponding strain. The determination of ultimate flexural capacity was predicated on yield of rebar steel, concrete failure, and the tensile yield of UHPFRC. However, the estimate of the moment in the initial cracking stage did not account for the contribution of the presently cracked NSC substrate. Figure 19 portrays the main details of an analytical model of a fortified slab.

The value of $x_{0}$ is obtained by the equilibrium forces in section (Figure 18): $T_{usb}+T_{ust}+T_{ub}=C_{u}$, as simplified in Equation (18).

(18) $$x_{0}=\frac{f_{y}A_{st}+f_{y}A_{sb}+f_{ut}bh_{1}}{b\left(f_{ut}+f_{u}\right)}$$

Accordingly, the predicted moment capacity of the reinforced slab underneath positive bending is estimated by carrying the moment of forces around the neutral axis $x_{0}$, as provided in Equation (19) below.

(19) $$M_{u}^{+}=\frac{f_{u}bx_{0}^{2}}{2}+\frac{f_{ut}b}{2}{\left(h_{1}-x_{0}\right)}^{2}+f_{y}A_{st}\left(h_{1}+a_{2}-x_{0}\right)+f_{y}A_{sb}\left(h-a_{3}-x_{0}\right)$$

$f_{u}\;and\;f_{ut}\;$denote compressive strength of UHPFRC and cracking strength, respectively; $A_{st}$ and $A_{sb}$ denote cross-section area of reinforcing steel in the NSC; $A_{su}$ denotes cross-section area of reinforcing steel in the UHPFRC.

Notwithstanding the numerous studies, these recommendations of analytical models require enhancement and development to encompass all newly proposed materials and combinations. Additionally, it is necessary to deliver a confirmed prediction of impact of critical loading on performance of fortified structure members, including dynamic and cyclic loads.

## 7. Recommendations for More Investigation

To improve knowledge of the performance of current RC members strengthened using the UHPFRC overlays, further investigations are recommended as follows:

1. Further studies are required to solidly review the provisions of the standards in the field of shaping the mixture, testing mechanical and strength properties and analytical calculations.
2. More studies are needed to develop a new bonding technique that assists in eliminating the issue of premature debonding.
3. The impact of the reinforcement steel rebars ratio in the UHPFRC jacket on the mode of failure requires additional investigation.
4. Numerous prior investigations focused on reinforcing the UHPFRC layer through steel reinforcement. Further studies must investigate the utilization of other reinforcing materials like CFRP rods to know the impact of these materials on the behavior of RC structures.
5. Most previous studies applied static loads to RC elements strengthened by UHPFRC layers. Further studies are required to examine the behavior of RC composite structures (slab/beam) under incremental repeated load (cyclic loading).
6. Additional research is required to investigate damaged reinforced concrete structures rehabilitation or strengthened by employing UHPFRC.
7. According to the observations from the FE model, a perfect bonding was often assumed at the interfacial contact model between NC and UHPFRC. New FEM approaches must be devised to enhance comprehension and prediction of fundamental contact behavior.

## 8. Conclusions

This investigation provides an exhaustive review of the current works on the rehabilitation or retrofitting of RC structural members utilizing UHPFRC. According to the evaluations of the practical results, FEM and analytical studies introduced in this article, here are some conclusions as follows:

1. The significantly reduced porosity and a denser microstructure of the UHPFRC enable enhanced durability. Consequently, it is beneficial for RC structures strengthened with UHPFRC to endure harsh conditions, including freeze–thaw cycles and the infiltration of chemical agents, particularly chlorides, which ultimately result in the corrosion of steel-reinforcing bars.
2. Experimental results demonstrate that UHPFRC may improve the flexural strength of members (slabs/beams) by 208%.
3. The most substantial enhancement in flexural strength occurred by beams reinforced with T-side UHPFRC (tensile side), owing to the beneficial strain-hardening characteristics of UHPFRC under tension.
4. This study reviewed three bonding approaches, all of which demonstrate a good performance overall.
5. The ideal thickness of the UHPFRC and steel fiber content may be 50 mm and 2%, respectively.
6. The experimental outcomes of the specimens demonstrate that the reinforcing of the UHPFRC layer with rebar steel has enhanced the maximum strength of the specimens by 119%.
7. Despite the increase in peak load, the issue of early debonding between the existing structures and additional strengthening layers still dominates most studies.
8. The results from finite element modeling closely aligned with experimental measurements, exhibiting variations between 0.93 and 1.06 for the ultimate load.

## Figures and Tables

**Table 1:** Typical composition of UHPFRC [29].

Components (kg/m^3^)	UHPFRC 200
Ordinary cement	955
Fine sand	1051
Fumes of silica	239
Superplasticizer	15
Steel fibers	168
Total water	162

**Table 2:** Experimental database.

Ref.	Specimen ID	Dimension	Bonding Technique	UHPFRC				*Pu* (kN)	*Du* (mm)	Failure Mode
				S. fibers vol. %	Configuration	Thickness (mm)	Reinforced			
[84]	RE-0	100 × 300 × 1600	/	/	/	/	/	61.08	14.78	*S*
	RE_ 20	100 × 300 × 1600	Cast in situ	3	T_ sided	20	/	57.18	28.52	*S* & *D*
	RE_ 32	100 × 300 × 1600	Cast in situ	3	T_ sided	32	Steel bar	43.68	34.65	*F*
	RE_ 50	100 × 300 × 1600	Cast in situ	3	T_ sided	50	Steel bar	55.38	25.68	*F*
	RE_ 100	100 × 300 × 1600	Cast in situ	3	/	100	/	113.05	19.09	*F*
	OV_ 25	125 × 300 × 1600	Cast in situ	3	T_ sided	25	/	73.47	14.70	*S* & *D*
	OV_ 25 a	125 × 300 × 1600	Cast in situ	3	T_ sided	25	Steel bar	77.97	14.42	*S*
	OV_ 50	150 × 300 × 1600	Cast in situ	3	T_ sided	50	/	77.97	9.44	*S* & *D*
	OV_ 50 a	150 × 300 × 1600	Cast in situ	3	T_ sided	50	Steel bar	95.06	17.76	*S*
[85]	NS	150 × 300 × 1584	/	/	/	/	/	250.84	4.63	*S*
	UNS2_ 1	150 × 300 × 1584	Cast in situ	1	T_ sided	150	Steel bar	436.24	6.15	*S*
	UNS2_ 1 D	150 × 300 × 1584	Dowel connection	1	T_ sided	150	Steel bar	402.25	5.55	*S*
	UNS2_ 1 S	150 × 300 × 1584	Stud connection	1	T_ sided	150	Steel bar	430.24	6.46	*S*
	UNS2_ 1.5	150 × 300 × 1584	Cast in situ	1.5	T_ sided	150	Steel bar	439.15	7.66	*S*
	UNS2_ 1.5 D	150 × 300 × 1584	Dowel connection	1.5	T_ sided	150	Steel bar	503.56	6.41	*S*
	UNS2_ 2	150 × 300 × 1584	Cast in situ	2	T_ sided	150	Steel bar	521.56	6.63	*S*
	UNS2_ 2 D	150 × 300 × 1584	Dowel connection	2	T_ sided	150	Steel bar	502.45	7.24	*S*
	HS	150 × 300 × 1584	/	/	/	/	/	256.61	4.62	*S*
	UHS2_ 1	150 × 300 × 1584	Cast in situ	1	T_ sided	150	Steel bar	528.41	8.57	*S*
	UHS2_ 1 D	150 × 300 × 1584	Dowel connection	1	T_ sided	150	Steel bar	439.22	9.52	*S*
	UHS2_ 1 S	150 × 300 × 1584	Stud connection	1	T_ sided	150	Steel bar	433.02	6.19	*S*
	UHS2_ 1.5	150 × 300 × 1584	Cast in situ	1.5	T_ sided	150	Steel bar	403.59	5.15	*S*
	UHS2_ 1.5 D	150 × 300 × 1584	Dowel connection	1.5	T_ sided	150	Steel bar	465.79	6.52	*S*
	UHS2_ 2	150 × 300 × 1584	Cast in situ	2	T_ sided	150	Steel bar	522.89	6.45	*S*
	UHS2_ 2 D	150 × 300 × 1584	Dowel connection	2	T_ sided	150	Steel bar	521.45	10.92	*S*
[86]	RC-Control	140 × 230 × 1600	/	/	/	/	/	70	19.10	*F*
	RC_ SB_ BOTSJ	140 × 260 × 1600	Cast in situ	2	T_ sided	30	/	81	15.31	*F* & *D*
	RC-SB-2SJ	200 × 230 × 1600	Cast in situ	2	2_ sided	30	/	102	13.38	*F*
	RC-SB-3SJ	200 × 260 × 1600	Cast in situ	2	3_ sided	30	/	132	4.55	*F*
	RC_ EP_ BOTSJ	140 × 260 × 1600	Epoxy adhesive	2	T_ sided	30	/	75	12.13	*F* & *D*
	RC-EP-2SJ	200 × 230 × 1600	Epoxy adhesive	2	2_ sided	30	/	95	15.7	*F*
	RC-EP-3SJ	200 × 260 × 1600	Epoxy adhesive	2	3_ sided	30	/	129	4.35	*F*
[87]	RCB	200 × 400 × 2600	/	/	/	/	/	246.56	31	*F*
	SPUB3–1	200 × 440 × 2600	Bolt connection	3	T_ sided	40	/	470.6	17	*P* & *D*
	SPUB3–2	200 × 440 × 2600	Bolt connection	3	T_ sided	40	/	448.7	10	*P* & *D*
	SPUB5–1	200 × 440 × 2600	Bolt connection	3	T_ sided	40	/	508	12	*P* & *D*
	SPUB5–2	200 × 440 × 2600	Bolt connection	3	T_ sided	40	/	484	10	*P* & *D*
	SPUB3	200 × 460 × 2600	Bolt connection	3	T_ sided	60	/	509.2	10	*P* & *D*
	RUB	200 × 460 × 2600	Cast in situ	3	T_ sided	60	Steel bar	449.8	8	*D*
[88]	CT_ 1.0	140 × 230 × 1120	/	/	/	/	/	383	2.49	*S*
	SB-2SJ_ 1.0	200 × 230 × 1120	Cast in situ	2	2_ sided	30	/	567	5.11	*S* & *F*
	SB-3SJ_ 1.0	200 × 260 × 1120	Cast in situ	2	3_ sided	30	/	628	4.19	*F*
	CT_ 1.5	140 × 230 × 1120	/		/		/	286	6.72	*S*
	SB-2SJ_ 1.5	200 × 230 × 1120	Cast in situ	2	2_ sided	30	/	402	8.96	*S* & *F*
	SB-3SJ_ 1.5	200 × 260 × 1120	Cast in situ	2	3_ sided	30	/	482	11.98	*F*
	CT_ 2.0	140 × 230 × 1120	/		/		/	276	8.68	*S*
	SB-2SJ_ 2.0	200 × 230 × 1120	Cast in situ	2	2_ sided	30	/	346	12.15	*S* & *F*
	SB-3SJ_ 2.0	200 × 260 × 1120	Cast in situ	2	3_ sided	30	/	353	12.26	*F*
[89]	P1	150 × 200 × 2200	/	/	/	/	/	55.2	11	*F*
	P2	150 × 200 × 2200	/	/	/	/	/	54.0	10.8	*F*
	U1	150 × 250 × 2200	Cast in situ	3	T_ sided	50	/	54.6	16	*D*
	U2	150 × 250 × 2200	Cast in situ	3	T_ sided	50	/	56.3	16.4	*S*
	UB1	150 × 250 × 2200	Cast in situ	3	T_ sided	50	Steel bar	102.1	12	*D*
	UB2	150 × 250 × 2200	Cast in situ	3	T_ sided	50	Steel bar	105.4	12.3	*S*
[90]	C_ S	150 × 300 × 2000	/	/	/	/	/	115	5.35	*S*
	C_ F	150 × 300 × 2000	/	/	/	/	/	253	15.77	*F*
	C_ S_ 210	210 × 300 × 2000	/	/	/	/	/	211	7.11	*S*
	ST_ 2S	210 × 300 × 2000	Epoxy adhesive	2	2_ sided	30	/	281	8.23	*F* & *D*
	ST_ 1S	210 × 300 × 2000	Epoxy adhesive	2	L_ sided	60	/	153	7.25	*S* & *D*
	ST_ 2S_ R	210 × 300 × 2000	Epoxy and Dowel	2	2_ sided	30	Steel bar	331	10.31	*F*
	ST_ 1S_ R	210 × 300 × 2000	Epoxy and Dowel	2	L_ sided	60	Steel bar	252	6.79	*F* & *S*
[91]	CB1	100 × 200 × 1500	/	/	/	/	/	69.62	28.04	*F*
	RA_ 1	100 × 210 × 1500	Epoxy adhesive	2	T_ sided	10	/	78.89	25.14	*F*
	RA_ 2	100 × 210 × 1500	Epoxy adhesive	2	T_ sided	10	/	75.32	23.31	*F*
	RA_ 3	100 × 210 × 1500	Epoxy adhesive	2	T_ sided	10	/	77.79	20.23	*F*
	RA_ 6	100 × 210 × 1500	Epoxy adhesive	2	T_ sided	10	/	78.84	22.53	*F*
	RB_ 1	100 × 210 × 1500	Epoxy adhesive	2	T_ sided	10	/	76.8	32.1	*F*
	RB_ 2	100 × 205 × 1500	Epoxy adhesive	2	T_ sided	5	/	76.15	23.46	*F*
	RB_ 3	100 × 208 × 1500	Epoxy adhesive	2	T_ sided	8	/	73.12	25.32	*F*
	RB_ 5	100 × 210 × 1500	Epoxy adhesive	2	T_ sided	10	/	84.6	29.39	*F*
	RB_ 6	100 × 212 × 1500	Epoxy adhesive	2	T_ sided	12	/	88.23	36.54	*F*
	RB_ 7	100 × 215 × 1500	Epoxy adhesive	2	T_ sided	15	/	93.21	40.12	*F*
	RB_ 8	100 × 210 × 1500	Epoxy adhesive	2	T_ sided	10	/	95.32	33.34	*F*
	RC_ 1	100 × 210 × 1500	Epoxy adhesive	2	T_ sided	10	/	82.74	21.34	*F*
	RC_ 2	100 × 210 × 1500	Epoxy adhesive	2	T_ sided	10	/	80.21	20.21	*F*
	RC_ 3	100 × 210 × 1500	Epoxy adhesive	2	T_ sided	10	/	84.12	24.93	*F*
[92]	RC_ 2.7	200 × 500 × 2700	/	/	/	/	/	655	/	*S*
	RC_ U_ 2.7	200 × 550 × 2700	Cast in situ	2.5	T-sided	50	/	717	/	*S* & *D*
	RC_ RU_ 2.7	200 × 550 × 2700	Cast in situ	2.5	T-sided	50	Steel bar	922	/	*S* & *D*
	RC_ RU_ 2.4	200 × 550 × 2400	Epoxy adhesive	2.5	T-sided	50	Steel bar	955	/	*S* & *D*
	RC_ RU_ 3.1	200 × 550 × 3100	Epoxy adhesive	2.5	T-sided	50	Steel bar	761	/	*S* & *D*
[93]	BL_ 0	250 × 400 × 3000	/	/	/	/	/	118.9	/	*F*
	BU_ 20	250 × 400 × 3000	Cast in situ	NM	T-sided	20	/	142.2	/	*F* & *D*
	BU_ 40	250 × 400 × 3000	Cast in situ	NM	T-sided	40	Steel bar	148.2	/	*F* & *D*
	BU_ 60	250 × 400 × 3000	Cast in situ	NM	T-sided	60	Steel bar	137	/	*F* & *D*
	BL_ 20	250 × 400 × 3000	Cast in situ	NM	T-sided	20	/	118.9	/	*F*
	BL_ 40	250 × 400 × 3000	Cast in situ	NM	T-sided	40	Steel bar	145.3	/	*F*
	BL_ 60	250 × 400 × 3000	Cast in situ	NM	T-sided	60	Steel bar	156.3	/	*F*
[94]	BEAM-1	150 × 250 × 3200	Control	/	/	/	/	43.21	14.31	*F*
	BEAM-2	150 × 300 × 3200	Epoxy adhesive	3	T-sided	50	/	67.97	15.81	*F* & *D*
	BEAM-3	150 × 300 × 3200	Bolt connection	3	T-sided	50	/	57.23	15.35	*F* & *D*
	BEAM-4	150 × 300 × 3200	Epoxy adhesive	3	T-sided	50	Steel bar	93.26	15.78	*F* & *D*
	BEAM-5	150 × 300 × 3200	Bolt connection	3	T-sided	50	Steel bar	89.23	52.29	*F* & *D*
	BEAM-6	150 × 300 × 3200	Epoxy adhesive	3	T-sided	50	Steel bar	109.63	19.08	*F* & *D*
	BEAM-7	150 × 300 × 3200	Bolt connection	3	T-sided	50	Steel bar	133.03	45.73	*F* & *D*

*Pu*: Ultimate load, *Du*: Deflection at maximum load, *S*: failure in shear, *D*: Debonding failure, *F*: Flexure failure, *P*: Peeling failure. NM: Not mentioned.

**Table 3:** Validation of the analytical models.

Ref.	Specimen ID	*EXP. Pu/FEM. Pu*
[150]	RE_20	0.95
	RE_32	1.02
	RE_50	1.06
	OV_25	1.03
	OV_25 a	0.95
	OV_50	0.94
	OV_50 a	1.00
[88]	CT_1.0	0.96
	SB_2SJ_1.0	0.94
	SB_3SJ_1.0	0.95
	CT_1.5	1.02
	SB_2SJ_1.5	1.01
	SB_3SJ_1.5	1.00
	CT_2.0	0.97
	SB_2SJ_2.0	1.02
	SB_3SJ_2.0	0.98
[89]	U1	0.94
	U2	0.94
	UB1	1.01
	UB2	1.01
[90]	C-S	1.02
	C-F	1.00
	C_S_21O	0.93
	ST_2S	1.03
	ST_1S	1.00
	ST_2S-R	1.01
	ST_1S-R	1.02
[91]	CBA	1.00
	RAA70	0.98
	RBB80	0.94
	RCC90	1.04

## Data Availability

All data generated or analyzed during this study are included in this published article.
